# Pathway analysis of body mass index genome-wide association study highlights risk pathways in cardiovascular disease

**DOI:** 10.1038/srep13025

**Published:** 2015-08-12

**Authors:** Xin Zhao, Jinxia Gu, Ming Li, Jie Xi, Wenyu Sun, Guangmin Song, Guiyou Liu

**Affiliations:** 1 Department of Cardiac Surgery, Qilu Hospital, Shandong University, Wenhuaxi Road, Jinan, China; 2 Department of Cardiology, The Fourth Affiliated Hospital of Harbin Medical University, Harbin, Heilongjiang,150001, China; 3 Department of Endocrinology and Metabolism, The Fourth Affiliated Hospital of Harbin Medical University. Harbin, Heilongjiang, 150001, China; 4 Genome Analysis Laboratory, Tianjin Institute of Industrial Biotechnology, Chinese Academy of Sciences, China

## Abstract

Cardiovascular disease (CVD) is a class of diseases that involve the heart or blood vessels. It is reported that body mass index (BMI) is risk factor for CVD. Genome-wide association studies (GWAS) have recently provided rapid insights into genetics of CVD and its risk factors. However, the specific mechanisms how BMI influences CVD risk are largely unknown. We think that BMI may influences CVD risk by shared genetic pathways. In order to confirm this view, we conducted a pathway analysis of BMI GWAS, which examined approximately 329,091 single nucleotide polymorphisms from 4763 samples. We identified 31 significant KEGG pathways. There is literature evidence supporting the involvement of GnRH signaling, vascular smooth muscle contraction, dilated cardiomyopathy, Gap junction, Wnt signaling, Calcium signaling and Chemokine signaling in CVD. Collectively, our study supports the potential role of the CVD risk pathways in BMI. BMI may influence CVD risk by the shared genetic pathways. We believe that our results may advance our understanding of BMI mechanisms in CVD.

Cardiovascular disease (CVD) is a class of diseases that involve the heart or blood vessels. It is reported that body mass index (BMI) is risk factor for CVD^1^. To evaluate the association between BMI and CVD mortality, Chen *et al.* conducted a pooled analysis of 20 prospective cohorts in Asia, including data from 835,082 East Asians and 289,815 South Asians^1^. The results showed that a high BMI is a risk factor for mortality from overall CVD and for specific diseases, including coronary heart disease, ischaemic stroke, and haemorrhagic stroke in East Asians^1^. In the United States, all major Hispanic/Latino groups have a high prevalence of obesity. Kaplan *et al.* analyzed the CVD and BMI data from US Hispanic men (N = 6547) and women (N = 9797)^2^. They found that elevated BMI is common in Hispanic/Latino adults and is associated with a considerable excess of CVD risk factors^2^.

Lamon-Fava *et al.* studied the distribution of BMI in men and women, and the association of BMI with known coronary heart disease (CHD) risk factors^3^. Their results indicate that increased BMI is associated with an adverse effect on all major CHD risk factors^3^. Loprinzi *et al.* examine the associations of underweight, overweight and obesity with kinds of CVD risk factors^4^. Their results showed that underweight adults had significantly lower levels of CRP; total cholesterol; total cholesterol to high-density lipoprotein cholesterol ratio; LDL cholesterol; and triglycerides compared to normal-weight individuals^4^. The overweight and obese adults had higher levels for each biomarker compared to normal-weight individuals^4^.

Much effort has been put into identifying the genetic determinants of CVD. Genome-wide association studies (GWAS) have recently provided rapid insights into genetics of CVD and its risk factors^5^,^6^,^7^,^8^,^9^,^10^,^11^. However, these newly identified susceptibility loci exert very small risk effects and cannot fully explain the underlying genetic risk. A large proportion of heritability has yet to be explained. Recent pathway analyses of GWAS have been put in the investigation of human disease pathogenesis and yielded important new insights into genetic mechanisms of human complex diseases, such as Alzheimer’s disease^12^,^13^, and rheumatoid arthritis^14^. Until now, the specific mechanisms how BMI influences CVD risk are largely unknown. We think that BMI may influences CVD risk by shared genetic pathways. In order to confirm this view, we conducted a pathway analysis of BMI GWAS, which examined approximately 329,091 single nucleotide polymorphisms (SNPs) from 4763 samples.

## Materials and Methods

### Study Population

The study subjects were from members of the Northern Finnish Birth Cohort of 1966 (NFBC1966)^15^. Mothers expected to give birth in the two Northern provinces of Oulu and Lapland in 1966 were enrolled in the NFBC1966 (N = 12058 live births, Rantakallio 1969)^15^. Primary clinical data collection on parents and the child occurred prenatal and at birth. Data collection on the child continued at ages six months, one year, 14 years (no data from one year or 14 years are included in this paper), 31 years, with assessment of a wide range of trait measures^15^. Informed consent from all study subjects was obtained using protocols approved by the Ethical Committee of the Northern Ostrobothnia Hospital District. The methods were carried out in accordance with the approved guidelines. Participants provided fasting blood samples for evaluation of the metabolic measures^15^. 4763 samples were genotyped using Illumina Infinium 370cnvDuo array^15^. According to the exclusion criteria and quality control procedures, SNPs were included for following analysis if the call rate in the final sample was >95%, if the P value from a test of Hardy-Weinberg Equilibrium (HWE) was >0.0001, and if the Minor Allele Frequency (MAF) was >1%^15^. In the end, 329,091 SNPs passed the quality controls and were selected for following analysis. For each SNP, the genotype was coded as 0, 1 or 2 copies of the minor allele. A regression analysis in PLINK was used to test the association between each SNP and BMI^15^.

### Gene-based testing for GWAS dataset

Here, we got the summary results from SNP-based test in the original study^15^ and performed a gene-based testing for GWAS dataset. ProxyGeneLD was used to assign SNPs to specific genes^16^. ProxyGeneLD begins with the retrieval of linkage disequilibrium (LD) structures in the HapMap genotyping data^16^. If a group of markers is in high LD in HapMap (r^2^ > 0.8), they are tied to a ‘proxy cluster’ and taken as a single signal^17^. Next, each marker in the BMI GWAS with statistically significant evidence of association is evaluated to see whether (a) it belongs to any proxy cluster and (b) whether the marker itself or any marker in the cluster is located in a genetic region^17^. If a marker or cluster overlaps a region extending across a gene, then it is assigned as showing possible association with that gene. Finally, a *P* value was given for each gene^16^. The *P* value was adjusted for the LD patterns in the human genome and gene length, but not multiple hypothesis testing correction. Genes with adjusted *P* < 0.05 are considered to be significant. For more detailed algorithms, please refer to the original study and our previous publications^16^.

### Pathway-based testing for GWAS dataset

The Kyoto Encyclopedia of Genes and Genomes (KEGG) pathways in WebGestalt were used^18^. For a given pathway, the hypergeometric test was used to detect the overrepresentation of BMI-related genes among all of the genes in the pathway^18^. The false discovery rate (FDR) method was used to correct for multiple testing. Any pathway with an adjusted *P* < 0.01 and at least five BMI genes was considered significant. In order to reduce the multiple-testing issue and to avoid testing overly narrow or broad pathways, we selected pathways that contained at least 20 and at most 300 genes for subsequent analysis.

## Results

### Gene-based test for GWAS dataset

We got 1008 significant BMI genes, which included 41 BMI genes with *P* < 0.001. TFAP2B is the most significant gene, which is reported to be significantly associated with BMI by previous studies^19^,^20^,^21^. Meanwhile, we identified other new BMI susceptibility genes. These genes were significantly associated with BMI with *P* < 0.001 (Table 1).

### Pathway-based analysis of GWAS dataset

We identified 31 significant KEGG pathways with at least five BMI genes. Based on the classifications of the KEGG pathways, these 31 pathways can be mainly divided into environmental information processing (n = 6), cellular processes (n = 5), circulatory system cellular processes (n = 5), metabolism (n = 5), endocrine system (n = 2), genetic information processing (n = 2), immune system (n = 2), nervous system and diseases (n = 2), cardiovascular diseases (n = 1) (Table 2). The detailed genes in these significant pathways are described in Supplementary Table 1.

## Discussion

In order to investigate how BMI influences CVD risk, we conducted a pathway analysis of BMI GWAS using 329,091 SNPs in 4763 Europeans. On the gene level, we identified 41 BMI genes with *P* < 0.001. TFAP2B is the most significant signal. Previous studies supported significant association of TFAP2B with BMI^19^,^20^,^21^. Speliotes *et al.* analyzed 249,796 individuals and revealed 18 new loci associated with BMI. The TFAP2B rs987237 variant is significantly associated with BMI with *P* = 3.00E-20^19^. Berndt *et al.* identified 11 new loci for anthropometric traits. The TFAP2B rs987237 variant is significantly associated with BMI (*P* = 2.00E-11)^20^. Wen *et al.* conducted a meta-analysis of GWAS in East Asian-ancestry populations and reported TFAP2B rs9473924 variant is significantly associated with BMI (*P* = 4.00E-07)^21^.

On the pathway level, we identified 31 significant KEGG pathways. Some of these pathways are identified to be associated with CVD. Here, we identified GnRH signaling pathway (hsa04912) to the second significant pathway. Sitras *et al.* conducted a gene expression profile analysis of CVD^22^. Gene set enrichment analysis showed significant association between GNRH signaling pathway and CVD^22^.

Vascular smooth muscle contraction (hsa04270) is the 5^th^ significant signal in our research. The vascular smooth muscle cell is a highly specialized cell. The principal function of vascular smooth muscle cell is contraction. By contraction, vascular smooth muscle cells shorten and decrease the diameter of a blood vessel to regulate the blood flow and pressure. Evidence shows that abnormal contraction of vascular smooth muscle is a major cause of vasospasm of the coronary and cerebral arteries^23^.

Here, we also highlighted the involvement of CVD related pathway in BMI. There are four CVD pathways in KEGG database, which include viral myocarditis (hsa05416), dilated cardiomyopathy (DCM) (hsa05414), hypertrophic cardiomyopathy (HCM) (hsa05410) and arrhythmogenic right ventricular cardiomyopathy (ARVC) (hsa05412). DCM is characterized by left ventricular dilation that is associated with systolic dysfunction^24^. In our research, we identified DCM to be significantly associated with AD with *P* = 3.30E-03.

There is also some literature evidence supporting the involvement of Gap junction, Wnt signaling, Calcium signaling and Chemokine signaling in CVD. More detailed information is described in Table 3.

Until now, there are kinds of software tools for pathway analysis of GWAS data^25^. Some tools including SNP ratio test^26^, GenGen^27^, GRASS^28^, and PLINK set-test^29^, accept raw genotype datasets as input data. Other tools including ProxyGeneLD^16^, ALIGATOR^25^, i-GSEA4GWAS^25^, and GESBAP^25^ accept the summary results to subsequent pathway analysis. Here, we selected ProxyGeneLD for gene-based test because we did not have access to raw CRC genotype data. This software adjusts for gene length and LD patterns in the human genome, which can reduce the sources of bias and increasing the reliability in pathway analysis. We selected the KEGG not the GO database for pathway analysis. It is reported that KEGG database is manually compiled based on biological evidence and does not have a hierarchical structure^30^,^31^, whereas the GO database is based on computer predictions and human annotation. It has a hierarchical structure^30^,^31^. GO analysis typically assumes that each functional category is independent, and less than 1% of the GO annotations have been confirmed experimentally^30^,^31^.

Despite these interesting results, we recognize some limitations in our study. Multiple testing corrections may not be sufficient to account for all biases in pathway analysis. The results from the BMI GWAS should be adjusted using a permutation test. However, the original SNP genotype data for each individual are not available to us now. When we get the SNP genotype data, we will further perform a pathway analysis using some available software such as SNP ratio test^26^, GenGen^27^, GRASS^28^, and PLINK set-test^29^. These pathway analysis methods or software can be used to analyze the SNP genotype data, and can conduct a permutation test. Future replication studies using genotype data are required to replicate our findings.

Collectively, our study supports the potential role of the CVD risk pathways in BMI. BMI may influence CVD risk by the shared genetic pathways. We believe that our results advance our understanding of BMI mechanisms in CVD and will be very informative for future genetic studies in BMI and CVD.

## Additional Information

**How to cite this article**: Zhao, X. *et al.* Pathway analysis of body mass index genome-wide association study highlights risk pathways in cardiovascular disease. *Sci. Rep.* **5**, 13025; doi: 10.1038/srep13025 (2015).

## Supplementary Material

Supplementary Table 1

## Figures and Tables

**Table 1:** The top 41 significant genes identified by gene-based analysis of BMI GWAS.

Gene ID	Gene Symbol	Gene Position	Unadjusted P	Adjusted P	Gene ID
7021	TFAP2B	chr6:50894397–50923285	rs987237	7.37E-07	4.42E-06
401036	ASB18	chr2:236768253–236837727	rs13386897	2.47E-06	1.97E-05
64222	TOR3A	chr1:177317734–177331752	rs6425512	1.27E-05	3.82E-05
4184	SMCP	chr1:151117421–151124147	rs3737861	2.10E-05	6.31E-05
9917	FAM20B	chr1:177261696–177312321	rs6425512	1.27E-05	6.37E-05
27	ABL2	chr1:177335084–177378834	rs6425512	1.27E-05	8.91E-05
6646	SOAT1	chr1:177529639–177591076	rs6425512	1.27E-05	1.40E-04
200186	CRTC2	chr1:152186775–152197667	rs11583896	1.55E-04	1.55E-04
23360	FNBP4	chr11:47694644–47745569	rs12286721	6.58E-05	1.97E-04
280636	C11orf31	chr11:57265297–57267459	rs9420	2.43E-04	2.43E-04
64225	ATL2	chr2:38376627–38457919	rs6760123	8.28E-05	2.48E-04
148327	CREB3L4	chr1:152207020–152213456	rs11583896	1.55E-04	3.10E-04
285051	C2orf61	chr2:47209090–47235930	rs1880583	4.09E-05	3.27E-04
64131	XYLT1	chr16:17103681–17472239	rs752482	6.97E-06	3.90E-04
79841	AGBL2	chr11:47637718–47692878	rs12286721	6.58E-05	3.95E-04
23279	NUP160	chr11:47756245–47826633	rs12286721	6.58E-05	3.95E-04
10658	CUGBP1	chr11:47446520–47467152	rs12286721	6.58E-05	3.95E-04
134353	LSM11	chr5:157103332–157116324	rs11738432	1.54E-04	4.61E-04
9909	DENND4B	chr1:152168600–152185778	rs11583896	1.55E-04	4.65E-04
4940	OAS3	chr12:111860631–111895438	rs10850109	1.17E-04	4.68E-04
54956	PARP16	chr15:63337489–63366071	rs894494	1.22E-04	4.86E-04
219541	MED19	chr11:57227762–57236249	rs9420	2.43E-04	4.86E-04
10237	SLC35B1	chr17:45133688–45140281	rs2289600	2.59E-04	5.17E-04
9717	SEC14L5	chr16:4948318–5009157	rs7191632	4.33E-05	5.63E-04
148022	TICAM1	chr19:4766938–4782737	rs16992609	2.85E-04	5.71E-04
56890	MDM1	chr12:66974612–67012428	rs10878810	9.97E-05	5.98E-04
2841	GPR18	chr13:98704967–98708683	rs7325747	3.12E-04	6.23E-04
26492	OR8G2	chr11:123600607–123601522	rs4936923	2.36E-04	7.09E-04
10978	CLP1	chr11:57181205–57185913	rs9420	2.43E-04	7.30E-04
51075	TXNDC14	chr11:57236617–57265020	rs9420	2.43E-04	7.30E-04
54974	THG1L	chr5:157090900–157099350	rs11738432	1.54E-04	7.69E-04
51368	TEX264	chr3:51680261–51713379	rs4974094	4.22E-04	8.44E-04
130827	TMEM182	chr2:102744921–102800570	rs2540289	1.44E-04	8.64E-04
84804	MFSD9	chr2:102700097–102719745	rs2540289	1.44E-04	8.64E-04
5493	PPL	chr16:4872508–4927137	rs1049206	7.23E-05	8.68E-04
57459	GATAD2B	chr1:152043826–152162075	rs11583896	1.55E-04	9.31E-04
3682	ITGAE	chr17:3564667–3651286	rs2891	7.27E-05	9.45E-04
25921	ZDHHC5	chr11:57192049–57225235	rs9420	2.43E-04	9.73E-04
1500	CTNND1	chr11:57285809–57343228	rs9420	2.43E-04	9.73E-04
3207	HOXA11	chr7:27187300–27191360	rs2189239	3.30E-04	9.90E-04
253832	ZDHHC20	chr13:20848507–20931423	rs4770145	1.25E-04	9.97E-04

**Table 2:** The significant KEGG pathways with *P* < 0.01 by pathway analysis of BMI GWAS.

Classifications	Pathway Name	Pathway ID	C	O	E	R	rawP	adjP
Endocrine system	Melanogenesis	hsa04916	101	17	2.36	7.22	2.00E-10	1.38E-08
Endocrine system	GnRH signaling pathway	hsa04912	101	13	2.36	5.52	6.89E-07	1.58E-05
Circulatory system	Gastric acid secretion	hsa04971	74	11	1.73	6.37	1.15E-06	1.98E-05
Nervous system	Long-term potentiation	hsa04720	70	10	1.63	6.12	5.10E-06	7.04E-05
Circulatory system	Vascular smooth muscle contraction	hsa04270	116	12	2.71	4.43	1.81E-05	2.00E-04
Cellular Processes	Oocyte meiosis	hsa04114	112	11	2.61	4.21	6.49E-05	3.00E-04
Cellular Processes	Gap junction	hsa04540	90	10	2.1	4.76	4.84E-05	3.00E-04
Circulatory system	Salivary secretion	hsa04970	89	10	2.08	4.82	4.39E-05	3.00E-04
Environmental Information Processing	Wnt signaling pathway	hsa04310	150	13	3.5	3.72	5.43E-05	3.00E-04
Environmental Information Processing	Calcium signaling pathway	hsa04020	177	14	4.13	3.39	7.73E-05	3.00E-04
Genetic Information Processing	Spliceosome	hsa03040	127	12	2.96	4.05	4.49E-05	3.00E-04
Immune system	Chemokine signaling pathway	hsa04062	189	15	4.41	3.4	4.18E-05	3.00E-04
Metabolism	Valine, leucine and isoleucine degradation	hsa00280	44	7	1.03	6.82	6.64E-05	3.00E-04
Metabolism	Purine metabolism	hsa00230	162	14	3.78	3.7	2.93E-05	3.00E-04
Cellular Processes	Lysosome	hsa04142	121	11	2.82	3.9	1.00E-04	4.00E-04
Cellular Processes	Phagosome	hsa04145	153	12	3.57	3.36	3.00E-04	1.10E-03
Metabolism	Histidine metabolism	hsa00340	29	5	0.68	7.39	5.00E-04	1.70E-03
Circulatory system	Pancreatic secretion	hsa04972	101	9	2.36	3.82	6.00E-04	2.00E-03
Metabolism	Propanoate metabolism	hsa00640	32	5	0.75	6.7	8.00E-04	2.50E-03
Cellular Processes	Endocytosis	hsa04144	201	13	4.69	2.77	9.00E-04	2.70E-03
Immune system	Complement and coagulation cascades	hsa04610	69	7	1.61	4.35	1.10E-03	3.20E-03
Cardiovascular diseases	Dilated cardiomyopathy	hsa05414	90	8	2.1	3.81	1.20E-03	3.30E-03
Circulatory system	Bile secretion	hsa04976	71	7	1.66	4.23	1.30E-03	3.40E-03
Environmental Information Processing	Hedgehog signaling pathway	hsa04340	56	6	1.31	4.59	1.90E-03	4.90E-03
Environmental Information Processing	Phosphatidylinositol signaling system	hsa04070	78	7	1.82	3.85	2.30E-03	5.70E-03
Genetic Information Processing	RNA transport	hsa03013	151	10	3.52	2.84	3.00E-03	7.10E-03
Environmental Information Processing	Jak-STAT signaling pathway	hsa04630	155	10	3.62	2.77	3.60E-03	7.80E-03
Infectious diseases: Parasitic	Amoebiasis	hsa05146	106	8	2.47	3.24	3.40E-03	7.80E-03
Metabolism	Lysine degradation	hsa00310	44	5	1.03	4.87	3.50E-03	7.80E-03
Environmental Information Processing	Cytokine-cytokine receptor interaction	hsa04060	265	14	6.18	2.26	4.10E-03	8.30E-03
Neurodegenerative diseases	Huntington’s disease	hsa05016	183	11	4.27	2.58	4.00E-03	8.30E-03

C, the number of reference genes in the category; O, the number of genes in the gene set and also in the category; E, expected number in the category; R, the ratio of enrichment, rawP, the p value from hypergeometric test; adjP, the p value adjusted by the multiple test adjustment.

**Table 3:** Literature evidence supporting pathways associated with CVD.

Pathway	Supporting evidence	Ref
Gap junction	Connexins, the protein molecules forming gap junction channels, are reduced in number or redistributed from intercalated disks to lateral cell borders in a variety of cardiac diseases	32
Gap junction	Alterations of gap junction organization and connexin expression are now well established as a consistent feature of human heart disease	33
Wnt signaling	Emerging evidence indicates that Wnt signaling regulates crucial aspects of cardiovascular biology (including cardiac morphogenesis, and the self-renewal and differentiation of cardiac progenitor cells).	34
Wnt signaling	Wnt signaling pathways play a key role in cardiac development, angiogenesis, and cardiac hypertrophy	35
Calcium signaling	Ca(2+)-related miRNAs have been found to be significant pathophysiological contributors in conditions like myocardial ischemic injury, cardiac hypertrophy, heart failure, ventricular arrhythmogenesis, and atrial fibrillation.	36
Chemokine signaling	There is growing evidence to suggest that chemokines play an important pathogenic role in cardiovascular diseases	37
