# Supplementary material for: Pathway analysis of body mass index genome-wide association study highlights risk pathways in cardiovascular disease
Source: Sci Rep. 2015 Aug 12;5:13025. doi: 10.1038/srep13025 (PMC4533004; doi:10.1038/srep13025)
Supplement: Supplementary Table 1 [file srep13025-s1.doc]

Supplementary Table 1, the detailed genes in significant pathways

| Pathway Name | Pathway ID | Gene |
| --- | --- | --- |
| Melanogenesis | hsa04916 | 2033 148327 196883 11211 1906 107 808 805 817 5579 4286 5443 5332 109 51384 5330 7484 |
| GnRH signaling pathway | hsa04912 | 10746 805 817 196883 5609 5579 107 808 109 30814 5332 3709 5330 |
| Gastric acid secretion | hsa04971 | 805 817 196883 5579 3772 107 808 5332 109 3709 5330 |
| Long-term potentiation | hsa04720 | 2033 805 817 5579 107 808 5332 10411 3709 5330 |
| Vascular smooth muscle contraction | hsa04270 | 805 196883 5579 107 808 109 30814 5332 5588 3709 136 5330 |
| Oocyte meiosis | hsa04114 | 805 817 196883 996 107 10971 808 109 3709 7532 29945 |
| Gap junction | hsa04540 | 10746 196883 5579 107 109 5332 79861 3709 5330 203068 |
| Salivary secretion | hsa04970 | 805 196883 486 5579 107 808 5332 109 3709 5330 |
| Wnt signaling pathway | hsa04310 | 2033 817 5579 10023 11211 166336 5332 51701 51384 5330 10297 4089 7484 |
| Calcium signaling pathway | hsa04020 | 805 817 6869 196883 5153 5579 22953 107 808 109 5332 3709 136 5330 |
| Spliceosome | hsa03040 | 6627 6632 23658 9879 84950 6431 51691 1655 6625 10907 988 11325 |
| Chemokine signaling pathway | hsa04062 | 2868 196883 6359 6368 5579 1445 59345 6358 6772 107 57580 109 6366 5332 5330 |
| Valine, leucine and isoleucine degradation | hsa00280 | 26275 217 5019 1962 4594 11112 223 |
| Purine metabolism | hsa00230 | 84284 5146 196883 5153 9061 107 109 5144 10623 5315 53343 5143 132 5430 |
| Lysosome | hsa04142 | 1511 968 1520 1497 2990 3074 23545 427 1513 3073 26985 |
| Phagosome | hsa04145 | 1781 1520 4689 4360 7060 23545 79861 3111 7099 529 8411 203068 |
| Histidine metabolism | hsa00340 | 51409 217 131965 3034 223 |
| Pancreatic secretion | hsa04972 | 196883 486 5579 107 30814 5332 109 3709 5330 |
| Propanoate metabolism | hsa00640 | 26275 217 1962 4594 223 |
| Endocytosis | hsa04144 | 2868 1601 390243 440073 55616 857 3560 51652 858 8027 8411 9815 84612 |
| Complement and coagulation cascades | hsa04610 | 1380 7450 3827 710 1378 735 5624 |
| Dilated cardiomyopathy | hsa05414 | 109 27091 196883 7170 4607 107 6444 9254 |
| Bile secretion | hsa04976 | 109 123264 196883 486 10599 107 2052 |
| Hedgehog signaling pathway | hsa04340 | 353500 51384 53944 64399 7484 51715 |
| Phosphatidylinositol signaling system | hsa04070 | 808 5332 805 3709 5330 5579 1608 |
| RNA transport | hsa03013 | 51095 55520 1967 91181 5905 1973 5901 10284 23279 100101267 |
| Jak-STAT signaling pathway | hsa04630 | 2033 1154 3600 56832 6772 2056 3447 3560 51561 8027 |
| Amoebiasis | hsa05146 | 1511 5579 107 5332 7099 5330 7414 735 |
| Lysine degradation | hsa00310 | 217 1962 9869 223 84444 |
| Cytokine-cytokine receptor interaction | hsa04060 | 6359 6368 115650 3600 56832 6358 23765 8743 6366 2056 3560 3447 4049 51561 |
| Huntington's disease | hsa05016 | 2033 4899 4698 148327 6908 3064 4722 3092 5332 5330 5430 |
